# Impact of diabetes on survival and clinical outcomes in elderly patients receiving peritoneal dialysis

**DOI:** 10.1080/0886022X.2025.2589586

**Published:** 2025-12-10

**Authors:** Luyao Xu, Yue Qian, Qianhui Song, Hao Yan, Zanzhe Yu, Zhenyuan Li, Na Jiang, Jiangzi Yuan, Jiaying Huang, Zhaohui Ni, Leyi Gu, Wei Fang

**Affiliations:** ^a^ Department of Nephrology, Renji Hospital, School of Medicine, Shanghai Jiao Tong University, Shanghai, People’s Republic of China; ^b^ Shanghai Center for Peritoneal Dialysis Research, Shanghai, People’s Republic of China

**Keywords:** Peritoneal dialysis outcomes, diabetes mellitus, elderly patients, end-stage renal disease (ESRD), mortality predictors, competing risk model

## Abstract

This study investigated the clinical outcomes and mortality risks in elderly peritoneal dialysis (PD) patients with diabetes mellitus (DM). A total of 210 incident PD patients aged 70 years or older (mean age 80.7 ± 5.0 years; 60.5% men) who initiated treatment between 2009 and 2020 were analyzed, including 81 (38.6%) with DM. Compared with non-diabetic patients, those with DM were younger (79.6 ± 4.7 vs. 81.4 ± 5.0 years, *p* = 0.011), and had higher fasting glucose (6.18 [4.53–9.30] vs. 4.88 [4.40–5.63] mmol/L, *p* < 0.001) and HbA1c (6.10% [5.43–6.78] vs. 5.40% [5.00–5.70], *p* < 0.001). Annualized mortality was 0.22 deaths per patient-year (95% CI 0.16–0.29) in the DM group and 0.20 (95% CI 0.16–0.25) in the non-DM group. Kaplan-Meier survival analysis revealed no significant differences in patient survival (*p* = 0.479), peritonitis-free survival (*p* = 0.953), or technique survival (*p* = 0.763) between the two groups in this cohort. In both Cox and Fine–Gray models, female sex (HR 1.505, 95% CI 1.033–2.193; *p* = 0.033) was an independent risk factor for all-cause mortality, whereas higher serum albumin (per 1 g/L increase: HR 0.938, 95% CI 0.906–0.971; *p* < 0.001) and higher residual kidney function (per 1 mL/min/1.73 m² increase: HR 0.881, 95% CI 0.808–0.960; *p* = 0.004) were independent protective factors. DM did not significantly predict mortality (HR 1.260, 95% CI 0.830–1.913; *p* = 0.279). These findings suggest that DM is not associated with worse clinical outcomes in elderly PD patients and may not represent a barrier to PD initiation.

## Introduction

The elderly patients constitute a substantial and growing fraction of the end-stage renal disease (ESRD) population [1,2]. According to the report of the United States Renal Data System (USRDS), the adjusted ESRD incidence in 2023 was 1581 per million population among individuals aged 75 years or older, representing the highest incidence across all age groups [1]. Similarly, the European Renal Association - European Dialysis and Transplant Association (ERA-EDTA) reported 55% of dialysis patients were 65 years or older in incident patients [3]. The elderly usually have a higher prevalence of comorbidities including diabetes mellitus (DM) [3,4]. For the elderly ESRD patients, peritoneal dialysis (PD) was one of the treatment options due to greater flexibility, fewer hemodynamic complications, and the convenience of home-based therapy, which can improve the quality of life for elderly patients [5,6]. It is generally accepted that diabetic PD patients have a worse survival, especially in elderly patients [7–9]. However, more series failed to find the difference in the outcome between diabetics and non-diabetics receiving PD treatment [10–12]. The impact of DM as a comorbidity on the prognosis of elderly PD patients is still controversial and whether DM is a contraindication for PD selection in older adults remains uncertain. Therefore, we conducted this study to investigate the association between outcomes and DM in elderly PD patients by comparing patient survival, peritonitis free survival and technique survival in diabetics and nondiabetics undergoing PD. We also analyzed the predictors of mortality in these two populations.

## Materials and methods

### Patients

All patients aged 70 years or older who initiated chronic PD at Renji Hospital, Shanghai Jiao Tong University School of Medicine, between 1 January 2009 and 31 December 2020 were screened for eligibility. We defined 70 as older, similar to the registry [13]. Exclusion criteria include patients with a history of maintenance hemodialysis (HD)/transplantation, withdrawal from PD within three months, or with critically incomplete data. All enrolled patients were dialyzed with lactate-buffered dextrose-based PD solutions (Dianeal^®^, Baxter). Our center is affiliated to a tertiary teaching hospital and is the largest PD program in Shanghai with over 800 PD patients being treated currently. The nurse-to-patient ratio is 1:120 at our center, and the APD proportion is 18%. All patients and/or their caregivers receive standardized training at PD initiation which remained stable throughout the 2009–2020 period. In accordance with international guidelines, a first-generation cephalosporin has consistently been the recommended antibiotic prophylaxis for PD catheter insertion over 20 years, with vancomycin reserved for allergic patients. This study was approved by the Human Research Ethics Committee of Renji Hospital, Shanghai Jiao Tong University School of Medicine. The requirement for written informed consent was waived because this was a retrospective study, and all personal information was securely protected and available only to the investigators.

### Data collection

Demographic characteristics collected at the time of enrollment included age, sex, height, weight, underlying etiology of ESRD, comorbidity status such as DM and cardiovascular disease (CVD), and self-care or assisted PD (PD exchanges performed by a family member or a domestic helper). DM was defined as patients with diabetic nephropathy as the primary renal disease and those with concurrent diabetes as a comorbidity. CVD was defined as a history of any of the following diseases: acute coronary syndrome, heart failure, atrial fibrillation, cerebral infarction or hemorrhage, percutaneous coronary intervention (PCI) or coronary artery bypass graft (CABG), or treatment-proven coronary atherosclerosis. Body mass index (BMI) was calculated as weight (kg) divided by the square of height (m) (BMI = weight [kg]/height [m^2^]). Baseline laboratory parameters were collected, including hemoglobin (HB), serum albumin, corrected calcium, phosphorus, intact parathyroid hormone (iPTH), total cholesterol, triglycerides, fasting blood glucose, glycated hemoglobin (HbA1c), high-sensitivity C-reactive protein (hs-CRP), and B-type natriuretic peptide (BNP). Corrected calcium (mmol/L) was calculated as total calcium (mmol/L) + (40 - albumin) × 0.025 (mmol/L). Small solute clearances and standard peritoneal equilibrium test (PET) were assessed 1–3 months after PD initiation in all enrolled patients. Small solute clearances were evaluated by collecting 24-h dialysate and urine and calculating weekly total Kt/V and weekly creatinine clearance (CrCl), normalized to 1.73 m^2^ body surface area. Residual kidney function (RKF) was measured as the average of 24-h urine urea and creatinine clearances. The normalized protein catabolic rate (nPCR) was calculated according to the method described by Randerson, Chapman, and Farrell, and standardized to standard body weight (total body water/0.58).

### Patients’ follow-up

All included patients were divided into diabetic (both with diabetic nephropathy as primary ESRD cause and those with diabetes as a comorbidity) and non-diabetic PD groups according to their comorbidity status and were followed up from PD initiation until death, transfer to permanent HD, PD decease due to recovery of kidney function, transfer to other center, loss to follow-up, or the end of the study (31 December 2021). All deaths, transfer to HD, and peritonitis episodes during the study period were carefully tracked and recorded. Causes of death and transfer to HD were also collected. Causes of death were divided into the following broad categories: cardiovascular (including cardiac, cerebrovascular, peripheral vascular, and sudden death); infections (including peritonitis and non-peritonitis infections); unknown and others. Causes of transfer to HD were categorized into peritonitis, mechanical complications of catheter (hernia and leakage), and other causes. Peritonitis was diagnosed and treated according to the International Society for Peritoneal Dialysis (ISPD) guidelines. Patient survival, peritonitis-free survival, and technique survival were calculated and compared between diabetic and non-diabetic elderly PD patients. For the analysis of patient survival and peritonitis-free survivals, the terminal events were death and the first peritonitis episode, respectively. The predictors of mortality between these two cohorts were analyzed. In the technique survival analysis, the terminal event was permanent switch to HD.

### Statistical analysis

Normally distributed data was presented as mean ± SD, while skew distributed data were described as median (interquartile range). Normally distributed continuous variables and skew distributed continuous variables were compared using the independent samples t-test and Mann-Whitney test, respectively. Additionally, the peritonitis rate (episode per patient-year) was compared between two groups by using Poisson regression. Categorical variables were expressed as frequencies and percentages and compared using chi-square tests. Missing data was handled by mean imputation for continuous variables. Survival curves for each relevant event (death, peritonitis, and transfer to HD) were estimated using the Kaplan–Meier and compared by Log-rank tests. Cox regression analyses were used to identify predictors of patient survival and peritonitis-free survival and to examine the role of potential confounders. Variables with *p* < 0.05 in the univariate analysis and important patients’ characteristics, except for multicollinearity, were entered into the multivariate analysis. The proportional hazards assumption for Cox models was assessed using Schoenfeld residuals (Supplement Figure 1). To ensure multivariable model stability, we evaluated the ratio of events to include 9 covariates for confirming adequacy given 134 deaths and calibration information to quantify model discrimination (Supplement Figure 2). Considering the presence of competing events in this study, for multivariate analysis, risk factors for all-cause mortality were evaluated by both cause-specific hazards and sub-distribution hazards models. When the event of interest was death, the competing events included transfer to HD, renal transplantation and transfer to other centers. Data was analyzed using the SPSS software package (version 27.0), R 4.5.1 and GraphPad Prism 10. All probabilities were two-tailed, and *p* < 0.05 was considered statistically significant.

## Results

### Patients’ characteristics

**Figure 1.**
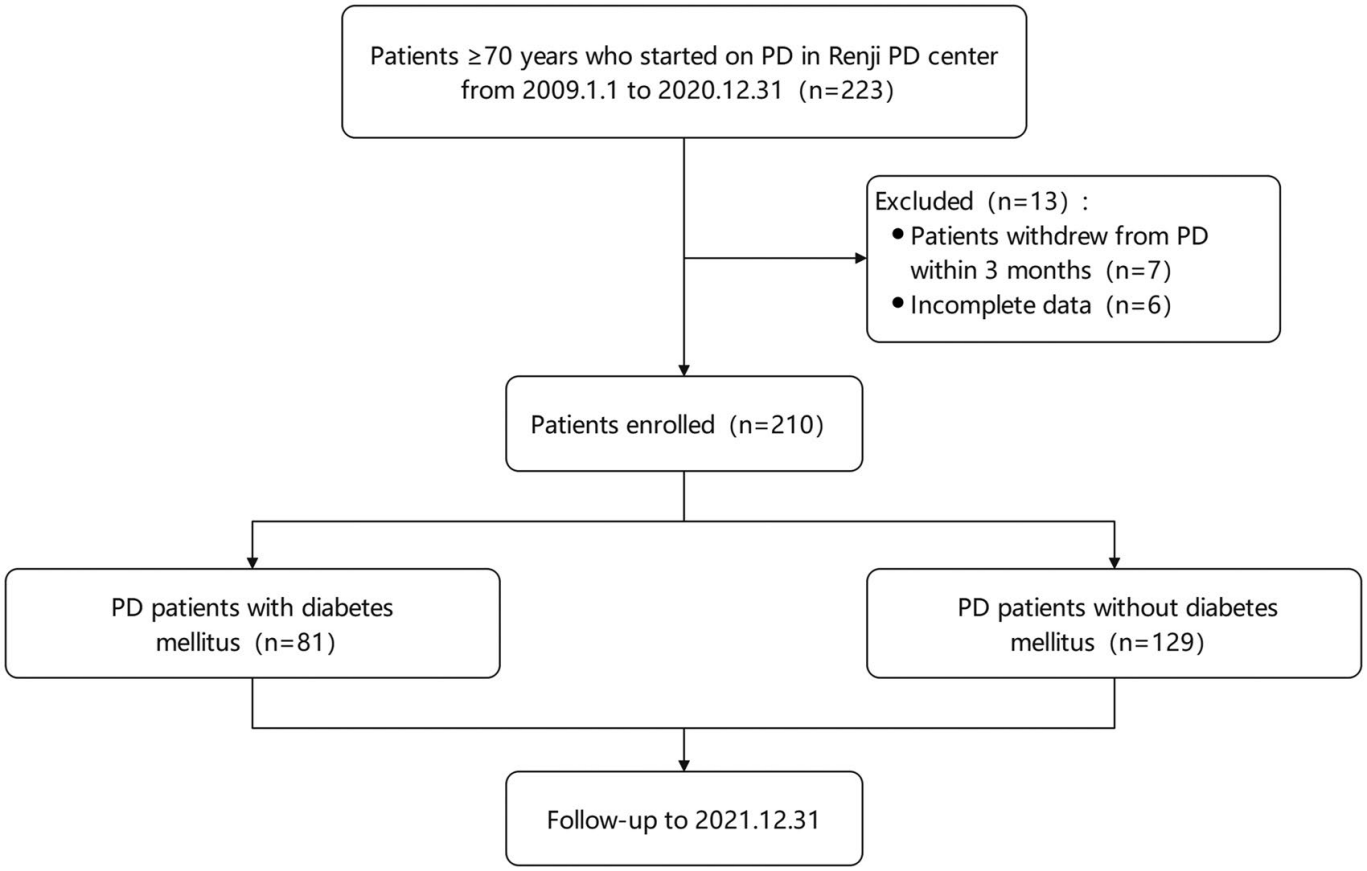
Patient enrollment and follow-up.

The study initially screened 223 patients. Among them, 13 patients were excluded due to various reasons. Finally, a total of 210 patients were included in the analyses (Figure 1). For the whole cohort, the mean age was 80.7 ± 5.0 years, 83 (39.5%) patients were female, 89 (42.4%) patients were on assisted PD, and 82 (39.0%) subjects were comorbid with CVD. Among the 210 patients, 81 (38.6%) patients were diabetics. Patients in the diabetic group were younger (79.6 ± 4.7 vs. 81.4 ± 5.0 years, *p* = 0.011), had higher fasting glucose levels (6.18 [4.53–9.30] vs. 4.88 [4.40–5.63] mmol/L, *p* < 0.001), and higher HbA1c levels (6.10% [5.43–6.78%] vs. 5.40% [5.00–5.70%], *p* < 0.001). Other demographic and laboratory data were similar between the two groups. No significant differences among small solute clearances, RKF, nPCR, and peritoneal transport characteristics (D/Pcr) were observed between patients from the two groups. Patient characteristics, small solute clearances and peritoneal transport characteristics are summarized in Table 1.

**Table 1.** Demographic, baseline laboratory, small solute clearances and peritoneal transport characteristics data of the study patients.

Variable	All PD patients (*n* = 210)	Non-diabetic group (*n* = 129)	Diabetic group (*n* = 81)	*p* value
Age (years)	80.7 ± 5.0	81.4 ± 5.0	79.6 ± 4.7	0.011*
Sex [*n* (%)]				0.775
Female	83 (39.5)	50 (38.8)	33 (40.7)	
BMI (kg/m^2^)	22.72 ± 3.39	22.51 ± 3.65	23.06 ± 2.93	0.230
Primary renal disease [*n* (%)]				0.001***
Chronic glomerulonephritis	64 (30.5)	50 (38.8)	14 (17.3)	
Diabetic nephropathy	40 (19.0)	0 (0.0)	40 (49.4)	
Hypertension	14 (6.7)	13 (10.1)	1 (1.2)	
Polycystic kidney disease	2 (1.0)	2 (1.6)	0 (0.0)	
Others	18 (8.6)	15 (11.6)	3 (3.7)	
Unknown	72 (34.3)	49 (38.0)	23 (28.4)	
Comorbidity [*n* (%)]				
Hypertension	184 (87.6)	109 (84.5)	75 (92.6)	0.083
Cardiovascular disease	82 (39.0)	50 (38.8)	32 (39.5)	0.914
Assisted PD [*n* (%)]				0.292
Self-care group	121 (57.6)	78 (60.5)	43 (53.1)	
Assisted group	89 (42.4)	51 (39.5)	38 (46.9)	
Baseline Laboratory results				
Hemoglobin (g/L)	110.2 ± 20.5	108.6 ± 21.4	112.9 ± 18.8	0.135
Albumin (g/L)	31.5 ± 5.6	31.4 ± 5.7	31.8 ± 5.4	0.626
Corrected calcium (mmol/L)	2.30 (2.18-2.42)	2.29 (2.18-2.41)	2.30 (2.17-2.43)	0.792
Phosphate (mmol/L)	1.29 (1.08-1.54)	1.30 (1.08-1.55)	1.28 (1.07-1.48)	0.669
Intact parathyroid hormone (iPTH) (pg/L)	149.0 (47.3-286.6)	144.0 (43.5-275.0)	165.5 (60.8-301.5)	0.509
Total cholesterol (mmol/L)	4.78 (4.05-5.65)	4.90 (4.11-5.73)	4.69 (4.00-5.39)	0.400
Total triglycerides (mmol/L)	1.40 (0.99-2.01)	1.36 (1.00-2.03)	1.45 (0.98-1.97)	0.234
Fasting blood glucose (mmol/L)	5.08 (4.41-6.48)	4.88 (4.40–5.63)	6.18 (4.53–9.30)	0.001***
Glycosylated hemoglobin (%)	5.50 (5.10-6.20)	5.40 (5.00-5.70)	6.10 (5.43-6.78)	0.001***
Hs-CRP (mg/L)	3.08 (1.14-8.12)	3.57 (1.21-8.82)	2.14 (0.94-6.12)	0.269
BNP (pg/ml)	161.5 (85.60-275.5)	167.0 (83.5-281.0)	158.0 (90.0-276.0)	0.364
Small solute clearances				
Total Kt/V urea	2.05 (1.74-2.43)	2.04 (1.73-2.40)	2.10 (1.74-2.53)	0.824
Total CrCl (L/week/1.73 m^2^)	67.45 (55.94-87.29)	65.14 (55.97-85.27)	71.47 (53.63-95.36)	0.579
RKF (mL/min/1.73 m^2^)	3.03 (1.73-4.95)	2.75 (1.58-4.82)	3.39 (1.88-5.37)	0.579
nPCR (g/kg/day)	0.83 ± 0.18	0.84 ± 0.19	0.80 ± 0.17	0.146
D/Pcr	0.66 ± 0.14	0.65 ± 0.13	0.67 ± 0.15	0.203

PD: Peritoneal Dialysis; RKF: Residual Kidney Function.

* *p* < 0.05.

** *p* < 0.01.

*** *p* < 0.001.

### Patients’ outcomes

After being followed up for 35.13 months (17.34–49.61 months), 134 (63.8%) patients had died, 21 (10.0%) had been transferred to HD, 9 (4.3%) had been transferred to other centers, 4 (1.9%) were no longer dialysis-dependent due to recovery of kidney function, 1 (0.5%) had been lost to follow-up, and 41 (19.5%) were still on PD. CVD was the leading cause of death in the whole elderly cohort. The median follow-up duration was 32.00 months (17.25–49.07 months) for the patients in diabetic group and 35.20 months (16.83–51.12 months) for their counterparts in non-diabetic group, respectively. Annualized mortality was 0.22 deaths per patient-year (95% CI 0.16–0.29) in the DM group and 0.20 (95% CI 0.16–0.25) in the non-DM group. The distributions of causes of death were similar between both groups (Table 2).

**Table 2.** Outcomes of the study patients.

Variable	All PD patients (*n* = 210)	Non-Diabetic group (*n* = 129)	Diabetic group (*n* = 81)	*p* value
Follow-up (months)	35.13 (17.34–49.61)	35.20 (16.83–51.12)	32.00 (17.25–49.07)	0.317
Outcomes [*n* (%)]				0.297
Death	134 (63.8)	83 (64.3)	51 (63.0)	
Transfer to HD	21 (10.0)	13 (10.1)	8 (9.9)	
Still on PD	41 (19.5)	24 (18.6)	17 (21.0)	
Lost to follow-up	1 (0.5)	1 (0.8)	0 (0.0)	
Transfer to other centers	9 (4.3)	4 (3.1)	5 (6.2)	
Recovery of renal function	4 (1.9)	4 (3.1)	0 (0.0)	
Causes of death [*n* (%)]				0.492
Cardiovascular disease	45 (33.6)	24 (28.9)	21 (41.2)	
Infection	36 (26.9)	25 (30.1)	11 (21.6)	
Others	37 (27.6)	24 (28.9)	13 (25.5)	
Unknown	16 (11.9)	10 (12.0)	6 (11.8)	
Causes of switch to HD [*n* (%)]				0.216
Peritonitis	9 (42.9)	7 (53.8)	2 (25.0)	
Catheter complications	5 (23.8)	1 (7.7)	4 (50.0)	
Cancer	2 (9.5)	1 (7.7)	1 (12.5)	
UFF	1 (4.8)	1 (7.7)	0	
Personal intention	2 (9.5)	2 (15.4)	0	
Unknown	2 (9.5)	1 (7.7)	1 (12.5)	
Peritonitis				
Total number of episodes	119	87	32	0.021*
Peritonitis rate (episode per patient-year)	0.183	0.210	0.136	0.033*
Peritonitis [*n* (%)]	76 (36.2)	48 (37.2)	28 (34.6)	0.588

PD: Peritoneal Dialysis; HD: Hemodialysis; UFF: Ultrafiltration Failure.

* *p* < 0.05.

** *p* < 0.01.

*** *p* < 0.001.

### Patient survival and predictors of all‑cause mortality

**Figure 2.**
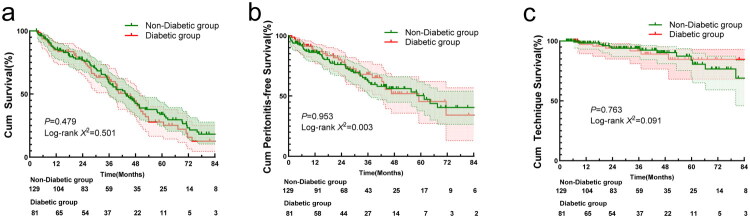
Kaplan–Meier curves for patients survival (a), peritonitis-free survival (b) and technique survival (c) in diabetic and non-diabetic elderly groups.

The patient survival in diabetic elderly patients was comparable to those of non-diabetics (Log-rank 0.501, *p* = 0.479) (Figure 2). In the multivariate Cox regression model, female sex (HR 1.505 [1.033–2.193]; *p* = 0.033) was an independent risk factor for all-cause mortality, whereas higher serum albumin (per 1 g/L increase: HR 0.938 [0.906–0.971]; *p* < 0.001) and higher RKF (per 1 mL/min/1.73 m² increase: HR 0.881 [0.808–0.960]; *p* = 0.004) were independent protective factors after adjusting for age, CVD, assisted PD or not, HB, and hs-CRP. Overall, risk factors were similar between the Cox and Fine-Gray models, with lower HB being significant only in the Fine-Gray analysis. However, for both models, no statistically significant difference in patient survival was detected between patients with and without DM in this elderly cohort (Table 3).

**Table 3.** Adjusted cs-HR (Cox model) and sd-HR (Fine and Gray model) analysis of independent risk factors for mortality in elderly PD patients.

	Increment	Cox model		Fine and Gray model	
		cs-HR (95% CI)	*p* value	sd-HR (95% CI)	*p* value
Age (years)	1 year	0.974 (0.932–1.017)	0.235	0.986 (0.946–1.029)	0.52
DM	–	1.260 (0.830–1.913)	0.279	1.183 (0.791–1.771)	0.41
Sex [*n* (%)]					
Male	–	Reference	Reference	Reference	Reference
Female	–	1.505 (1.033–2.193)	0.033*	1.545 (1.084–2.203)	0.016*
Comorbidity [*n* (%)]					
Cardiovascular disease	–	1.210 (0.828–1.768)	0.325	1.251 (0.866–1.808)	0.23
Assisted PD [*n* (%)]					
Self-care group	–	Reference	Reference	Reference	Reference
Assisted group	–	1.280 (0.836–1.960)	0.256	1.244 (0.810–1.909)	0.32
Baseline Laboratory results					
Hemoglobin (g/L)	1 g/L	0.991 (0.982–1.000)	0.057	0.989 (0.980–0.998)	0.018*
Albumin (g/L)	1 g/L	0.938 (0.906–0.971)	0.001***	0.949 (0.917–0.981)	0.002**
Hs-CRP (mg/L)	1 mg/L	1.005 (0.992–1.019)	0.432	1.007 (0.997–1.018)	0.15
RKF (mL/min/173 m^2^)	1 mL/min	0.881 (0.808–0.960)	0.004**	0.860 (0.793–0.932)	0.001***

PD: Peritoneal Dialysis; DM: Diabetes Mellitus; RKF: Residual Kidney Function.

* *p* < 0.05.

** *p* < 0.01.

*** *p* < 0.001.

We further examined the predictors of all-cause mortality in diabetic and non-diabetic elderly patients respectively. In the diabetic group, higher serum albumin (per 1 g/L increase: HR 0.935 [0.878–0.996]; *p* = 0.038) and higher RKF (per 1 mL/min/1.73 m² increase: HR 0.838 [0.716–0.981]; *p* = 0.028) were independent protective factors of mortality. In the non-diabetic group, female sex (HR 1.806 [1.102–2.959]; *p* = 0.019) and assisted PD (HR 2.178 [1.211–3.919]; *p* = 0.009) were independent risk factors associated with death, whereas higher serum albumin (per 1 g/L increase: HR 0.940 [0.898–0.983]; *p* = 0.007) and higher RKF (per 1 mL/min/1.73 m² increase: HR 0.884 [0.793–0.984]; *p* = 0.024) were independent protective factors (Table 4).

**Table 4.** Multivariate Cox regression analysis of predictors of mortality in diabetic and non-diabetic groups.

		Non-Diabetic group		Diabetic group	
	Increment	HR (95% CI)	*p* value	HR (95% CI)	*p* value
Age (years)	1 year	0.969 (0.914–1.028)	0.303	0.961 (0.892–1.034)	0.284
Sex [*n* (%)]					
Male	–	Reference	Reference	Reference	Reference
Female	–	1.806 (1.102–2.959)	0.019*	1.106 (0.607–2.012)	0.743
Comorbidity [*n* (%)]					
Cardiovascular disease	–	1.008 (0.617–1.646)	0.975	1.512 (0.772–2.962)	0.228
Assisted PD [*n* (%)]					
Self-care group	–	Reference	Reference	Reference	Reference
Assisted group	–	2.178 (1.211–3.919)	0.009**	0.652 (0.354–1.203)	0.171
Baseline Laboratory results					
Hemoglobin (g/L)	1 g/L	0.993 (0.982–1.004)	0.191	0.989 (0.971–1.007)	0.217
Albumin (g/L)	1 g/L	0.940 (0.898-0.983)	0.007**	0.935 (0.878-0.996)	0.038*
Hs-CRP (mg/L)	1 mg/L	1.004 (0.988–1.021)	0.612	1.010 (0.985–1.035)	0.450
RKF (mL/min/1.73 m²)	1 mL/min	0.884 (0.793–0.984)	0.024*	0.838 (0.716–0.981)	0.028*

PD: Peritoneal Dialysis; RKF: Residual Kidney Function.

* *p* < 0.05.

** *p* < 0.01.

*** *p* < 0.001.

### Peritonitis and peritonitis‑free survival

During the study period, 76 (36.2%) patients experienced their first episode of peritonitis, with 28 (34.6%) in the diabetic group and 48 (37.2%) in the non-diabetic group. As shown in Figure 2, the peritonitis-free survival for elderly patients with DM was comparable to that of patients without DM (Log-rank 0.003, *p* = 0.953). Both univariate and multivariate Cox regression analyses revealed no significant association between the presence or absence of DM and peritonitis-free survival in elderly PD patients. Additionally, no variables were found to be significantly associated with peritonitis-free survival in this cohort (*p* > 0.05).

### Technique survival

There was no significant difference in technique survival between diabetic and non-diabetic elderly patients (Log-rank 0.091, *p* = 0.763) (Figure 2). Given that only 21 (10.0%) patients in this cohort had been transferred to HD, including 8 (9.9%) diabetic patients, Cox regression analysis was not performed due to the small number of events.

## Discussion

In the present study, we compared the outcomes of PD patients aged 70 years or older with or without DM to investigate the impact of diabetes as a comorbidity on the overall prognosis of elderly patients undergoing PD. Our results showed that, in the elderly PD patients, diabetic patients had similar patient survival, peritonitis-free survival and technique survival to their non-diabetic counterparts. No statistically significant difference in patient survival was detected between patients with and without DM in this elderly PD cohort. This study provides one of the largest single-center analyses of PD outcomes among adults 70 years or older, demonstrating that DM loses its prognostic impact in this age group, challenging traditional contraindications for PD in diabetic ESRD.

There were minor differences in the demographic and clinical characteristics of the elderly PD patients between diabetic and non-diabetic groups. As expected, the diabetic elderly patients showed higher fasting blood glucose and HbA1c levels compared to the non-diabetic group. Patients in the diabetic group were younger than those in the non-diabetic group. Perhaps it is because diabetes-induced renal function deterioration progresses more rapidly, potentially leading to the need for dialysis at a relatively younger age [14,15].

The causes of death in diabetic elderly PD patients were similar to non-diabetics. It is well known that CVD is the most common cause of deaths in PD patients [1,16]. In the present study, CVD remained the leading cause of mortality among elderly PD patients, accounting for 33.6% of all deaths. Additionally, infection was also a major cause of mortality, accounting for 26.9% of deaths, similar to the results from a prospective study of elderly PD patients [1]. This result suggested that older PD patients were prone to infection, might be due to a high prevalence of comorbidity, physical disability, poor nutrition and immunodeficiency, which is common in elderly ESRD patients. Our finding underscores the need to aggressively prevent and treat both cardiovascular complications and infections when managing elderly PD patients. Peritonitis was the primary reason for permanent switch to HD, accounting for 42.9% of cases, which is consistent with the reports by Francis et al. [4] and Shamspour et al. [17]. Again, the critical importance of preventing peritonitis in elderly PD patients has been highlighted.

In terms of patient survival, whether DM adversely affects outcomes remains controversial. Previous studies have demonstrated that patients’ survival is comparable or slightly better in the early years after dialysis initiation for PD compared with HD, whereas long-term survival tends to be poorer in PD patients, particularly among those with diabetes [18,19]. A study involving 5802 PD patients found the survival advantage of PD diminished with increasing patient age [20]. Meanwhile, a registry based study included 18,110 PD patients showed that diabetic PD patients aged 55 years and older had higher risks of all-cause mortality [21]. However, our study found that, in patients aged 70 or older, there is no statistically significant survival difference between diabetic and non-diabetic patients. Our finding is consistent with the results reported by Lamping et al. in the North Thames Dialysis Study [22]. They found that, in 221 ESRD patients aged 70 years or over, DM was not associated with patients’ death. Recently Gao et al. [23] reported markedly different 1-, 3- and 5- year survival between elderly Chinese subgroups, which did not identify DM as an independent risk factor affecting patient survival. Both Cox and FG model identified female sex, lower serum albumin, and lower RKF as independent predictors of mortality. Elderly female patients generally likely related to the potential loss of cardioprotective effect from estrogen deficiency and present with more frailty [24], which has been identified as an independent predictor of mortality in PD populations. And they have lower muscle mass and higher fat mass compared with males [25], which may adversely affect nutritional status. It has been shown that, compared to male patients, female PD patients have a higher incidence of peritonitis and dialysis catheter infections [26], which may partly explain the higher mortality observed among female elderly PD patients in our study. Hypoalbuminemia, as a marker of malnutrition and inflammation, is a well-established predictor of mortality in PD patients [27], and preservation of RKF remains critical strategy for improving survival [28], which is also applicable to the elderly PD population. In this context, nutritional optimizations such as adequate protein intake, management of inflammation, and timely treatment with comorbidity and complication may help reduce mortality risk. Likewise, strategies aimed at preserving RKF, including avoiding nephrotoxic agents, maintaining adequate volume control, and minimizing ultrafiltration stress, are particularly important in elderly patients, in whom RKF is often fragile. These findings underscore the importance of targeting modifiable factors such as nutrition and RKF preservation to improve outcomes in elderly PD patients. Importantly, DM was not identified as a mortality risk factor in present elderly PD cohort. Firstly, we speculated that elderly dialysis patients may be a highly selected group and more motivated subgroup with generally better health or less severe disease, especially diabetic elderly PD patients. As it is plausible that patients with severe diabetic complications may have been considered unsuitable for PD [29] or died before reaching dialysis initiation, which remains a hypothesis and warrants further investigation. Moreover, in older patients, the prognostic impact of DM may be attenuated by survival bias, competing cardiovascular and infectious mortality. And elderly diabetic patients typically receive closer clinical monitoring and more aggressive management of blood glucose and cardiovascular risk factors, which could partially mitigate adverse effects [30]. Finally, PD itself offers better hemodynamic stability than hemodialysis, which may benefit diabetic patients with cardiovascular comorbidities and reduce modality-related risks. Our result suggested that in elderly ESRD patients, diabetes may not represent a barrier to selecting PD treatment.

We found the predictors of mortality varied a little between diabetic and non-diabetic elderly cohorts. While lower serum albumin and lower RKF were common risk factors, female sex and assisted PD were also associated with death in the non-diabetic group. The slight variation in mortality predictors highlights the importance of developing customized stratification and management plans for diabetic and non-diabetic elderly patients. But we recognize that subgroup analyses, particularly for the diabetic cohort (*n* = 81), may have limited power to detect some associations, potentially resulting in type II errors.

Although the peritonitis rate was significantly higher in the elderly non-diabetic group, there was no significant difference in peritonitis-free survival between elderly diabetic and non-diabetic patients. This aligns with findings from a cohort study of 351 patients with peritonitis from 1979 to 2014, which showed that the previously reported increased risk of peritonitis in diabetic patients is no longer evident in recent years [31]. One multicenter observational study involving 114 dialysis centers [32] and the other multicenter observational study involving 57 dialysis centers [12] also reported DM was not a risk factor for the first episode of peritonitis. For clarify, we have included a small comparative table summarizing 5 large cohorts (Table 5). In our study, differences in patient characteristics between the two groups were minimal, which may partially explain the comparable peritonitis-free survival. Additionally, improvements in PD training and infection control measures may have mitigated the increased peritonitis risk traditionally associated with diabetes [33]. Diabetic patients, often considered high-risk, might receive more intensive monitoring and education. Despite comparable peritonitis-free survival, peritonitis remains a major contributor to adverse outcomes in PD patients. This highlights the importance of strengthening patient education, support, and management, alongside implementing continuous quality improvement (CQI) programs [34], which have proven effective in reducing peritonitis rates.

**Table 5.** Summary of key large cohort studies on diabetes and outcomes in PD patients.

First author and Year	Registry/Region	Sample size	Population	Study design	Main findings regarding DM/outcomes
Lamping et al. [22]	North Thames (UK)	221	Dialysis patients ≥70 years	Prospective cohort	No significant survival difference between diabetic and non-diabetic elderly dialysis patients.
Martin et al. [32]	BRAZPD (Brazil)	2032	Incident PD patients	Prospective cohort	Age >65 and DM were not independent predictors of first peritonitis
See et al. [13]	ANZDATA (Australia & New Zealand)	16,748	Incident PD patients	Cohort / competing-risk regression	Age >70 and DM were predictors of early technique failure, but DM no associated with death.
Chen et al. [7]	ANZDATA (Australia & New Zealand)	8279	Adults initiating PD	Retrospective cohort / registry	Diabetic nephropathy (DN) associated with higher mortality and technique failure; effect more pronounced in women.
Ljungman et al. [12]	PEPS (Peritonitis Prevention Study, Sweden)	671	Incident PD patients	Prospective multicenter cohort	DM was not identified as an independent predictor of time to first peritonitis.

PD: Peritoneal Dialysis; DM: Diabetes Mellitus.

Technique survival was also similar between elderly diabetic and non-diabetic PD patients in our study. This is consistent with studies by Cotovio et al. [35], Meng et al. [36], and Bonenkamp et al. [37], which found no significant difference in PD technique survival between diabetic and non-diabetic patients. Our study supports these findings, reinforcing that DM may not represent a barrier to initiating PD in elderly patients.

Our study has several limitations. Firstly, it was conducted at a single center with a homogeneous cohort of elderly Chinese patients, which may limit the generalizability of our findings to other populations. Differences in ethnicity, comorbidity profiles, dialysis practices, and health care systems may lead to different outcomes in other settings. However, our study provides more detailed data on patients’ comorbidities and laboratory findings, with all patients receiving care from the same medical team, minimizing inter-center confounding factors. Secondly, as a retrospective cohort study, potential biases and confounding factors cannot be entirely excluded. The grouping of patients with diabetic nephropathy as the primary ESRD cause and those with diabetes as a comorbidity, despite showing no significant outcome differences in our analysis, may have diluted associations specific to each group. Thirdly, we did not collect data regarding the quality of life (QoL), hospitalization rates, and cardiovascular event incidence, which are equally important endpoints for elderly patients receiving PD. Clearly, prospective studies with larger sample sizes and multi-center participation are warranted.

These findings support PD candidacy among elderly diabetic patients, inform shared decision-making, and may guide guideline revisions on PD eligibility in frail or comorbid populations.

In summary, the outcomes of elderly diabetic and non-diabetic PD patients were comparable. Therefore, DM may not represent a barrier to initiating peritoneal dialysis in elderly ESRD patients.

## Supplementary Material

Supplemental Material

Supplemental Material

## Data Availability

The data that support the findings of this study are not publicly available due to privacy restrictions but are available from the corresponding author upon reasonable request.
