# Supplementary figures and images for: Impact of diabetes on survival and clinical outcomes in elderly patients receiving peritoneal dialysis
Source: Ren Fail. 2025 Dec 10;47(1):2589586. doi: 10.1080/0886022X.2025.2589586 (PMC12697273; doi:10.1080/0886022X.2025.2589586)

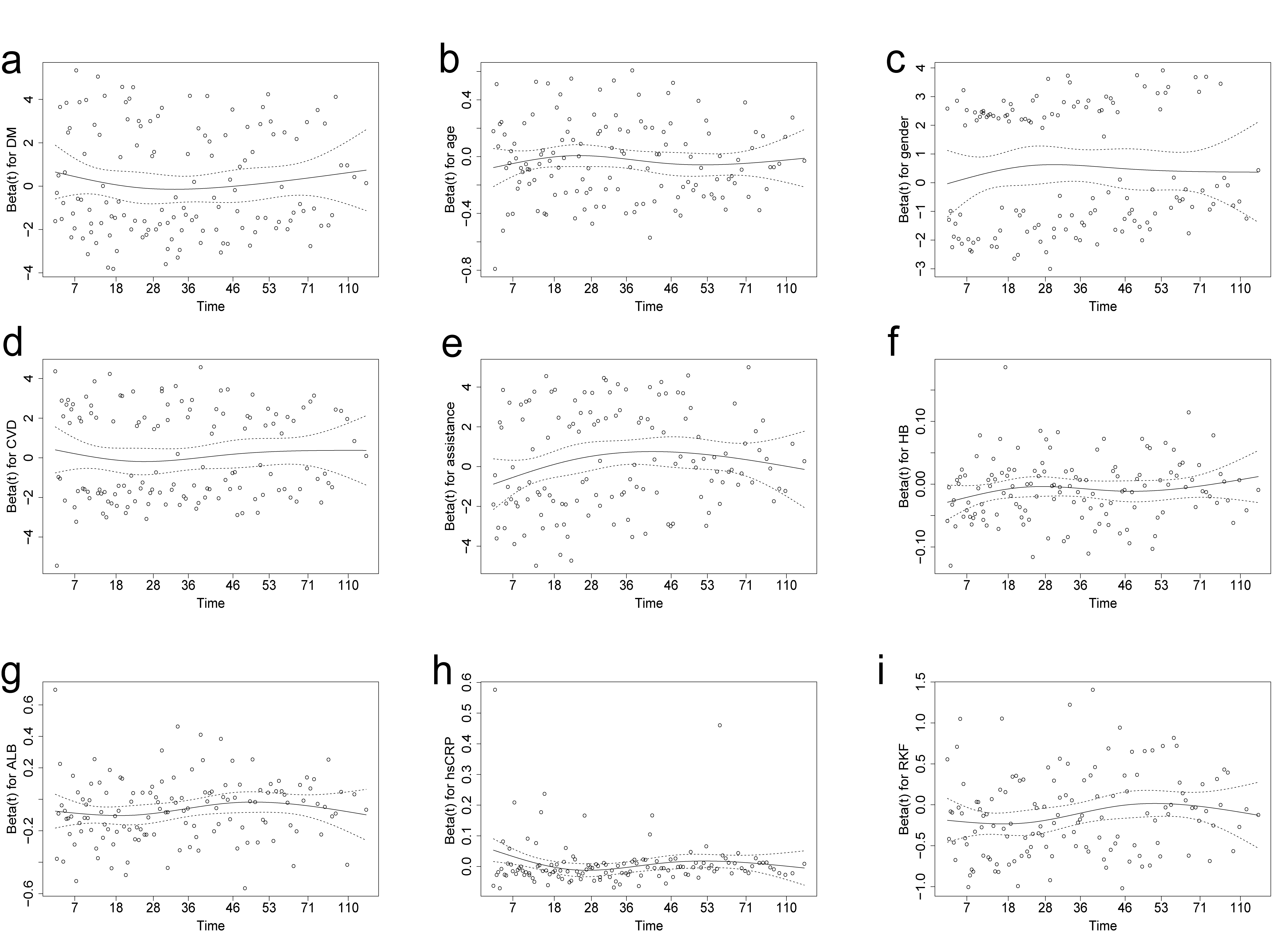

Supplement: Supplemental Material [file IRNF_A_2589586_SM5008.tif]

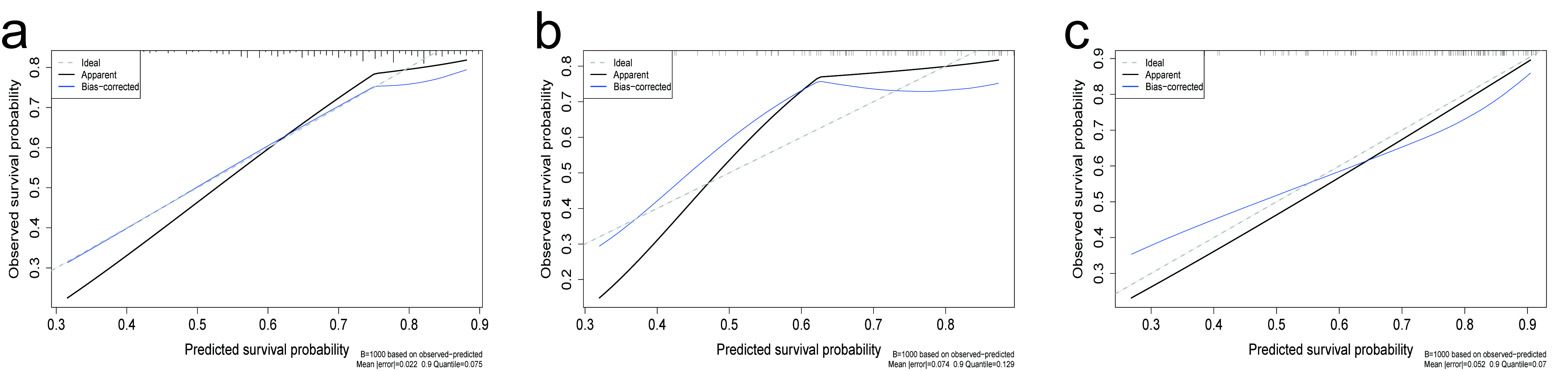

Supplement: Supplemental Material [file IRNF_A_2589586_SM4998.tif]
